# Purpura thrombopénique amégacaryocytaire acquis: penser au lupus érythémateux systémique

**DOI:** 10.11604/pamj.2015.20.86.5694

**Published:** 2015-01-29

**Authors:** Indretsy Mahavivola Ernestho-ghoud, Odilon Rahamefy, Irina Mamisoa Ranaivo, Malalaniaina Andrianarison, Lala Soavina Ramarozatovo, Fahafahantsoa Rapelanoro Rabenja

**Affiliations:** 1 Unité de Soins de Formation et de la Recherche en Dermatologie, Centre Hospitalier Universitaire Joseph Raseta Befelatanana, Antananarivo, Madagascar

**Keywords:** Thrombopénie, myélogramme, amegacaryocytose acquise, lupus érythémateux systémique, Thrombopenia, myelogram, Acquired amegakaryocytosis, SLE

## Abstract

L'amegacaryocytose acquise est exceptionnellement décrite au cours d'un Lupus Erythémateux Systémique (LES) à Madagascar. Nous rapportons la première observation d'un Purpura Thrombopénique Amegacaryocytaire Acquis (PTAA) simulant un Purpura Thrombopénique Idiopathique (PTI) révélateur d'un LES. Il s'agissait d'une femme de 24 ans, sans antécédents particuliers. Elle présentait un syndrome hémorragique avec une thrombopénie à 10 000/mm^3^. Le diagnostic de PTI était retenu avant l'hospitalisation. Elle avait reçu une corticothérapie mais ceci n’était pas suivi d'amélioration. A l'unité de Dermatologie, elle se plaignait d'une baisse de l'acuité visuelle. Elle était en bon état général. On retrouvait une tachycardie à 110 bpm, un érythème malaire en verspertilio typique et une pâleur cutanéo-muqueuse. Une hémorragie oculaire bilatérale était objectivée à l'examen ophtalmologique. Les examens paracliniques montraient une thrombopénie à 31000/mm^3^, une anémie microcytaire à 48g/dL. Les examens immunologiques étaient non réalisés. Un LES avec atteinte cutanée et hématologique était retenu. Un bolus de corticothérapie était administrée associée à une transfusion sanguine. L’évolution était marquée par l'apparition d'un signe d'engagement cérébral faisant suspecter un neurolupus. Le scanner cérébral révélait une hémorragie cérébrale avec une hydrocéphalie aigue traitée par un inhibiteur de l'anhydrase carbonique mais le neurolupus n’était pas écarté. L'anémie disparaissait par contre la thrombopénie s'aggravait à 16000/mm^3^. Le médullogramme montrait l'absence des mégacaryocytes. L’évolution était favorable à huit mois de suivi après un relais per os de la corticothérapie par la dose de 1 mg/kg/j à dose dégressive à huit mois de suivi. Les atteintes neurologiques, ophtalmologiques et hématologiques étaient compatible avec le diagnostic d'un LES. La persistance d'une thrombopénie doit faire suspecter une amegacaryocytose. Le myélogramme était indispensable pour poser le diagnostic

## Introduction

L'amegacaryocytose acquise est une affection rare, caractérisée par une thrombopénie sévère et une amégacaryocytose médullaire [1]. A notre connaissance, elle n'a jamais été décrite au cours d'un lupus érythémateux systémique (LES) à Madagascar. Elle pouvait être confondue avec un authentique purpura thrombopénique idiopathique (PTI) causant des erreurs diagnostiques. Nous rapportons la première observation d'une patiente présentant un purpura thrombocytopenique amegacaryocytaire acquis (PTAA) révélant un LES.

## Patient et observation

**Figure 1:**
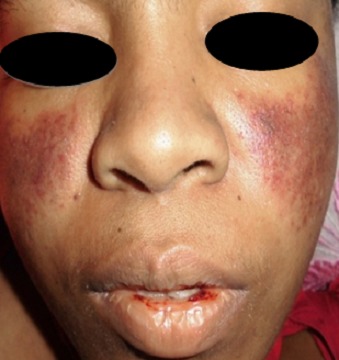
Erythème malaire typique du visage et un érythème péribuccal évoquant une vascularite

Il s'agissait d'une patiente âgée de 24 ans, ayant présenté un syndrome hémorragique (un purpura pétéchial, une épistaxis, une gingivorragie et une menometrorragie) évoluant depuis Janvier 2014. L'hémogramme avant l'hospitalisation révélait une thrombopénie à 10000/mm^3^. Le diagnostic de PTI était retenu avant l'hospitalisation.- Une corticothérapie à la dose de 1mg/kg/j était instituée pour une durée de dix jours mais sans amélioration. Elle était référée à l'unité de Dermatologie du CHU de Befelatanana. Aucune notion d'exposition aux irradiations ionisantes ni de prise de toxiques n'avait été signalée. Elle n'avait pas d'antécédents personnels ni familiaux particuliers. Elle se plaignait d'une baisse brutale de l'acuité visuelle. A l'admission, l'examen physique retrouvait une patiente en bon était général, une pâleur cutanéo-muqueuse, une tachycardie à110bpm et- un érythème malaire en verspertilio du visage typique (Figure 1) et un érythème péribuccal évoquant une vascularite. L'examen ophtalmologique objectivait une acuité visuelle à 1/10 bilatérale, une quadranopsie bilatérale temporale inférieure gauche et nasale droite. Le tout était en faveur d'une hémorragie rétinienne maculaire et retro-hyloïdienne. Le reste de l'examen clinique était normal. L'investigation paraclinique montrait une anémie microcytaire à 48g/dL, une thrombopénie à 31000/mm^3^, une vitesse de sédimentation à 65 mm, une C reactive protein à 12 mg/L. La protéinurie de 24h était revenue négative. Le bilan immunologique (anticorps antinucléaire, DNA natif, anticorps anticytoplasmes des polynucléaires neutrophiles et anticorps antiplaquettes) étaient non faits pour un problème pécuniaire. Le diagnostic d'un LES avec atteinte cutanée et hématologique était retenu. Une corticothérapie en bolus à la dose de 15mg/kg/j pendant 3 jours était administrée associée à une transfusion sanguine de sang total.

**Figure 2:**
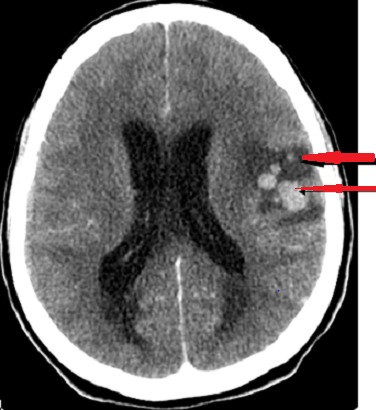
Multiples opacités spontanément hyperdense de taille différentes (petite flèche) et œdème périlésionnel (grande flèche) en faveur d'une hémorragie cérebro-méningée multiple avec une inondation du 4 eme ventricule compliquée d'une hydrocéphalie aiguée

Aux deuxièmes jours, elle présentait une confusion mentale, une crise convulsive d'emblée généralisée, une raideur de la nuque et un signe de Babinski bilatéral évoquant un signe d'engagement cérébral. Ce tableau neurologique faisait suspecter un neurolupus. - La tomodensitométrie cérébrale sans injection de produit de contraste (Figure 2) révélait des multiples opacités spontanément hyperdense de taille différentes (petite flèche) et œdème périlésionnel (grande flèche) en faveur d'une hémorragie cérebro-méningée multiple avec une inondation du 4 eme ventricule compliquée d'une hydrocéphalie aiguée. L'image scannographique cérébrale était douteuse au début pouvant faire suspecter une neuroparasitose ou une métastase cérébrale d'une tumeur solide. L’échographie pelvienne ainsi que la radiographie du thorax ne retrouvaient pas d'images suspectes d'une malignité. Mais devant ce tableau neurologique, l'association d'une thrombopénie avec un syndrome hémorragique extériorisé à type de purpura et menometrorragie, un hématome cerebral était retenu. Le traitement institué associait un inhibiteur de l'anhydrase carbonique pour une durée de dix jours et poursuite des corticoïdes à 1mg/kg/j. Au bout de trois jours, une hémiparésie gauche s'installait, se récupérant spontanément évoquant un vasospasme. Par contre, l'anémie disparaissait et la thrombopénie s'aggravait à 16000/mm^3^. Le myélogramme montrait l'absence des mégacaryocytes. Le diagnostic d'un LES avec atteinte cutané et hématologique était retenu. L'amegacaryocytose acquise était responsable d'une hémorragie cutanée et viscérale (ORL, gynécologique, ophtalmologique et en particulier cérébrale) responsable des manifestations neurologiques mais un neurolupus ne pouvait pas être éliminé. L’évolution était favorable sous corticothérapie à la dose de 1mg/kg/j à dose dégressive avec un état général correct, une disparition du syndrome hémorragique, une amélioration de l'acuité visuelle et une normalisation de l'hémogramme à huit mois de contrôle.

## Discussion

Il s'agissait d'une première observation médicale d'un LES révélé par un PTAA parmi 86 cas de LES recensé dans le service de Dermatologie du CHU d'Antananarivo sur une période de 11 ans allant de 2002 à 2013. Le LES est une affection auto-immune, caractérisé par des atteinte multi systémiques de gravité variable. Les atteintes neuropsychiatriques constituent un signe de gravité et un facteur de mauvais pronostic. Le diagnostic de neurolupus reste compliquer en l'absence de marqueurs diagnostiques fiables pour chaque type de manifestation clinique. Il doit toujours être posé devant un faisceau d'arguments chronologiques, cliniques et paracliniques [2]. Pour notre cas, l'hémorragie cérébrale était au premier plan. Le neurolupus ne pouvait pas être éliminé formellement. Il faut y penser chez le sujet jeune du fait de sa gravité. Par ailleurs, l'atteinte hématologique au cours du LES est fréquente, pouvant engager le pronostic vital dans certaines situations. En 2010, certains auteurs constatent que les autres manifestations associées à une atteinte hématologique du LES sont l'anémie hémolytique, les convulsions et la vascularite cérébrale. La thrombopénie est associée à la lymphopénie, à la neutropénie et à la survenue d'une atteinte oculaire du LES [3]. Certains de ces signes étaient cités chez notre patiente telle que l'anémie, la convulsion et l'atteinte oculaire résultant toutes de la complication de la thrombopénie. Initialement, l'atteinte hématologique (purpura associé à un syndrome hémorragique) et une thrombopénie évoque un PTI [4]. Les manifestations cliniques d'un PTAA sont semblables à celles d'un PTI [1]. La confusion diagnostique est donc possible entre ces deux affections, ce qui doit inciter, en cas de doute, à faire un myélogramme pour déterminer l'origine périphérique en cas de PTI, avec des mégacaryocytes normaux voire élevés au myélogramme [4]. Le diagnostic d'un PTAA repose à la biopsie osteomédullaire qui montre l′absence de mégacaryocytes, alors que les précurseurs granuleux et érythroïdes sont normaux [1]. Les causes de PTAA sont nombreuses incluant les infections virales, toxiques, médicamenteuses et le LES [5]. Une thrombopénie secondaire à la corticothérapie n'a pas encore été décrite. L'association d'une anémie, une thrombopénie amegacaryocytaire écartait aussi un syndrome d'Evans. L’élément essentiel permettant de retenir le LES était l’érythème malaire en verspertilio typique du visage en l'absence de causes toxiques ou d'expositions aux irradiations ionisantes. Il était fort probable que le LES était la cause de PTAA chez notre patiente. En effet, le PTI posait un réel problème de diagnostic différentiel avec un authentique PTAA. Le mécanisme d'un PTAA au cours LES est encore mal connu. La dernière hypothèse évoquée est la présence d'un autoanticorps antirecepteur de la thrombopoeitine, qui bloque les signaux de la thrombopoeitine et freine la thrombopoeiese, responsable de l'amegacaryocytogenese [6, 7]. La confirmation d'un PTAA permet donc de prévenir leurs complications telles qu'une myelodysplasie voire une aplasie médullaire [5] et d’éviter le recours à des thérapeutiques lourdes de PTI (Corticoïdes, Immuglobulines polyvalentes et surtout splénectomie). Par contre, différents traitements sont utilisés de façon empirique au cours d'un PTAA avec de degré d'efficacité variable. En 2004, les Glucocorticoïdes sont rapportés efficaces de façon isolée [8]. Des essais des facteurs de croissances plaquettaires en 2010 (Eltrombopag ^®^) [9], l'anti-CD20 (Rituximab ^®^) en 2014 [10], sont efficaces au cours d'un PTAA sur un LES réfractaire aux traitements. Pour notre observation, le diagnostic du LES avec atteinte cutanée, hématologique et neurologique était évident malgré la confirmation par les bilans immunitaires. La Corticothérapie par voie générale était efficace chez notre patiente sous réserve de l’évolution dans le temps.

## Conclusion

Une thrombopénie au cours d'un lupus érythémateux systémique n’était pas toujours d'origine périphérique. Un myélogramme s'impose pour éliminer une amegacaryocytose acquise car exposait la patiente à une myelodysplasie voire une aplasie médullaire. Le LES est l'une des causes d'une amegacaryocytose acquise, méritant d’être connu et recherché.
